# Diagnostic utility of serum TRACP5b for secondary osteoporosis in ankylosing spondylitis: a comparative cross-sectional study with primary osteoporosis and healthy controls

**DOI:** 10.1007/s10067-026-07966-7

**Published:** 2026-02-17

**Authors:** Doaa Kamal, Nihal Fathi, Asmaa Osama B. S. Osman, Amira Mohammad Abdalmageed, Marwa A. A. Galal

**Affiliations:** 1 Rheumatology, Rehabilitation and Physical Medicine Department, Faculty of Medicine, Assiut University, Assiut, Egypt; 2 Clinical Pathology and Immunology, Faculty of Medicine, Assiut University, Assiut, Egypt

**Keywords:** Ankylosing spondylitis, TRACP5b, Osteoporosis

## Abstract

**Objectives:**

To evaluate serum tartrate-resistant acid phosphatase 5b (TRACP5b) levels in patients with ankylosing spondylitis (AS) and primary osteoporosis (OP), and to assess its diagnostic value, correlations with bone mineral density (BMD) and disease activity, and its potential as a marker of secondary osteoporosis in AS.

**Methods:**

In this cross-sectional comparative study, 23 patients with AS, 24 patients with primary OP, and 20 healthy controls were recruited from Assiut University Hospitals. Demographic, clinical, laboratory, and dual-energy X-ray absorptiometry (DXA) data were collected. AS disease activity was assessed using Bath Ankylosing Spondylitis Disease Activity Index (BASDAI). Serum TRACP5b was measured by ELIZA. Group differences, correlations, and ROC curve analysis were performed.

**Results:**

AS patients were predominantly male and younger than OP patients. Primary OP patients had significantly lower DXA T-scores than AS patients (mean difference = 2.36, *p* < 0.001). TRACP5b levels were higher in AS patients than controls but not significantly different (*p* = 0.111). In AS, TRACP5b correlated with age (*r* = 0.534, *p* = 0.009) and disease activity (*r* = 0.427, *p* = 0.042) but not with BMD. ROC analysis showed moderate diagnostic performance for detecting secondary osteoporosis in AS (AUC = 0.653). No significant differences in TRACP5b or BMD were found across AS treatment groups.

**Conclusion:**

Serum TRACP5b may serve as a supplementary marker of osteoclast activity in AS and shows moderate diagnostic value for secondary osteoporosis, with levels more related to age and disease activity than BMD. Larger studies are needed to confirm its clinical utility.

**Table:** 

**Key Points** • *Serum TRACP5b levels were higher in patients with AS than in healthy controls, though without consistent statistical significance, reflecting biological variability.* • *TRACP5b correlated positively with age and disease activity (BASDAI) in AS but not with BMD, disease duration, or ESR, highlighting its link to inflammatory bone resorption.* • *ROC analysis showed TRACP5b had moderate diagnostic performance for secondary osteoporosis in AS, but limited value in primary osteoporosis.* • *Our findings suggest that TRACP5b may serve as supplementary marker of bone turnover in AS, warranting further validation in larger, longitudinal studies.*

**Supplementary Information:**

The online version contains supplementary material available at 10.1007/s10067-026-07966-7.

## Introduction

Osteoporosis (OP) is a condition characterized by increased bone fragility due to reduced bone mineral density (BMD) and/or microarchitectural deterioration, both of which elevate fracture risk [1]. Osteoporosis is broadly classified as either primary or secondary, depending on the underlying factors affecting bone metabolism. Primary osteoporosis includes involutional osteoporosis type I (postmenopausal osteoporosis, due to estrogen deficiency) and type II (senile osteoporosis) [2]. Secondary osteoporosis can be caused by various diseases such as ankylosing spondylitis (AS), certain medications, and lifestyle factors [3]. Dual-energy X-ray absorptiometry (DXA) remains the gold standard for assessing BMD and diagnosing OP. According to World Health Organization (WHO) guidelines, osteoporosis is defined as a T-score ≤ −2.5, while osteopenia is defined as a T-score between −1.0 and −2.5 [4]. However, DXA provides a static quantitative measure of areal bone mass and does not capture dynamic changes in bone turnover or metabolic activity. Consequently, DXA alone may not fully reflect ongoing bone remodeling process, particularly in conditions characterized by inflammation-driven alterations in bone metabolism [5].

As is an autoinflammatory rheumatic disease that predominantly affects the axial skeleton. Its pathogenesis involves chronic inflammation and disruption of normal bone homeostasis, resulting in both aberrant new bone formation and concurrent bone loss [6]. Reduced vertebral BMD can be observed even in the early stages of AS and is associated with an increased risk of vertebral fractures and progressive spinal deformity [7, 8].

Several recent studies have highlighted the value of plasma biomarkers in monitoring AS activity, disease progression, bone turnover and in guiding therapeutic decisions [9]. Tartrate-resistant acid phosphatase (TRACP) is a glycosylated metalloprotein acid phosphatase, expressed by bone-resorbing osteoclasts, inflammatory macrophages, and dendritic cells [10]. Two isoforms circulate in human blood: TRACP5a, primarily derived from macrophages, and dendritic cells, and TRACP5b, which is produced directly by osteoclasts and reflects osteoclast number and activity [11]. TRACP5b has gained attention as a specific bone resorption marker that is unaffected by renal function [12]. Elevated TRACP5b levels have been linked to enhanced bone resorption and increased fracture risk in various populations [13]. Its role as a biomarker of bone remodeling has been explored in various inflammatory and rheumatic diseases, including AS [11, 14].

Given the impact of secondary osteoporosis in AS and the limitations of DXA in capturing inflammation-related bone changes, there is a need to explore complementary biomarkers that reflect bone metabolic activity. Therefore, this study aimed to evaluate serum TRACP5b levels in patients with primary osteoporosis and secondary osteoporosis due to AS, to compare BMD and disease-related characteristics among studied groups, and to assess the diagnostic utility and clinical correlations of TRACP5b in patients with AS.

## Materials and methods

This was a cross-sectional comparative analytical study conducted at the Rheumatology, Rehabilitation, and Physical Medicine Department, Assiut University Hospitals. Adults aged ≥ 18 years were consecutively assessed for eligibility. A total of 75 individuals were screened, of whom 8 were excluded leaving 67 participants for final analysis. They were categorised into 3 groups: 23 patients diagnosed with AS according to the modified New York criteria [15], 24 patients with primary OP confirmed by DXA, and 20 apparently healthy controls who were age- and sex-matched with the AS group. No matching was performed between the control group and the primary OP group, due to the demographic characteristics of the latter. Exclusion criteria included the presence of other autoimmune or connective tissue diseases or failure to meet AS or OP diagnostic criteria (4 participants), systemic glucocorticoids use within the preceding 6 months (1 participant), or secondary causes of OP unrelated to AS (e.g., endocrine, gastrointestinal, or renal disorders) (1 participant). Individuals who declined to participate or did not provide informed consent (2 participants) were also excluded.

All participants underwent detailed clinical evaluation, including demographic history (age, sex, and smoking status) and relevant medical history including disease duration, medications use with dosing and duration, and history of fragility fractures. Each participant received a comprehensive physical and rheumatological examination, and disease activity in AS patients was assessed using the Bath Ankylosing Spondylitis Disease Activity Index (BASDAI) [16]. Venous blood samples were collected for detecting complete blood count (CBC), liver and kidney function tests, and serum tartrate-resistant acid phosphatase 5b (TRACP5b). Human TRACP5b levels were measured by human enzyme-linked immunosorbent assay (ELISA) commercial kit manufactured by Wuxi donglin Sci and Tech Development Co., Ltd. (Dldevelop), USA. Blood samples were centrifuged, and serum was stored at −20°C until analysis, following the manufacturer's instructions.

Bone mineral density (BMD) was assessed at the standard anatomical sites (lumbar spine L1-L4 anteroposterior view, left or right femoral neck, left or right wrist) using the Lunar DPX DXA system manufactured by GE Healthcare.

### Statistical analysis

Sample size estimation was performed a priori using G*Power software version 3.1 [17]. Based on an expected effect size (f = 0.72) for differences in serum TRACP5b levels between groups, with a two-tailed α of 0.05 and a statistical power of 95%, a minimum sample size of 33 participants (11 per group) was required. To account for potential exclusions or incomplete data, the target sample size was increased, ultimately, 67 participants were included, exceeding the calculated minimum.

Statistical analyses were conducted using Jamovi version2.6.2 for macOS (The Jamovi Project). Continuous variables were presented as medians with interquartile ranges or means with standard deviations, as appropriate; categorical variables were expressed as frequencies and percentages.

Comparisons between the groups were performed using the Wilcoxon rank-sum test for continuous variables and Pearson’s Chi-square test for categorical variables. Correlations were assessed with Pearson’s correlation coefficients, and ROC curve analysis was used to evaluate the diagnostic accuracy of TRACP5b, reporting AUC, sensitivity, specificity, and optimal cut-offs.

Statistical significance was set at *p* ≤ 0.05. Effect sizes and confidence intervals were reported where relevant.

## Results

A total of 47 patients were included in the study: 23 with AS and 24 with primary OP, in addition to 20 apparently healthy controls. Among the AS patients, 18 were males (78%) and 5 were females (22%), with a median range of 37.0 years (range: 33.0–43.8). The primary OP group included 4 males (17%) and 20 females (83%), with a median age of 71.0 years (range: 69.4–75.0). The control group consisted of 15 males (75%) and 5 females (25%), with a median age of 37.0 years (range: 29.1–40.0). Age differed significantly across the three groups (Kruskal–Wallis F₂,₆₄ = 73.75, *p* < 0.01). Gender distribution also showed significant difference between groups (Pearson’s χ^2^ = 22.53, *p* < 0.01).

### Comparison of disease-related variables

AS patients had a significantly longer disease duration compared to primary OP patients (*p* < 0.01). Smoking prevalence was also significantly higher in the AS group (*p* = 0.03). There was no significant difference in the history of fragility fractures between the two groups (Table 1).

**Table 1:** Comparison of disease-related variables between patients with AS and primary OP

	AS	Primary OP	Test Statistic	*p*-value
	(*N* = 23)	(*N* = 24)		
**Disease Duration**	11.0 (7.2 −16.5)	0.5 (0.5–0.5)	F_1,45_ = 291.87	P < 0.01^3^
**Smoking**			χ^2^₂ = 7.31	P = 0.03^2^
**Ex smoker**	2 (8.7%)	2 (8.3%)		
**No**	15 (65.2%)	22 (91.7%)		
**Smoker**	6 (26.1%)	0 (0%)		
**History Of Fragility Fracture: No**	23 (100%)	23 (95.8%)	χ^2^₁ = 0.98	P = 0.32^2^

Data are presented as median [range] for continuous variables or N (%) for categorical variables. AS ankylosing spondylitis, OP osteoporosis. ^1^Kruskal-Wallis. ^2^Pearson. ^3^Wilcoxon. *P* ≤ 0.05 was considered statistically significant.

### Bone mineral density

**Fig. 1:**
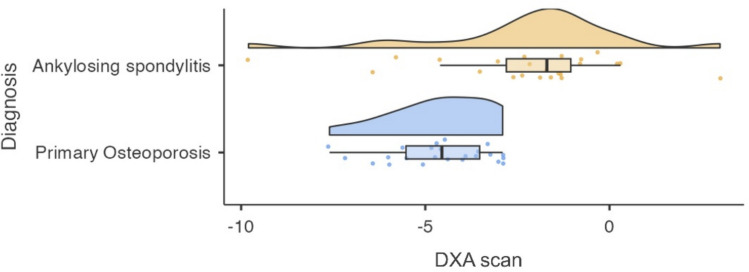
Violin plot showing the distribution of DXA T-scores in patients with AS and primary OP

Patients with primary OP had significantly lower DXA T-scores than those with AS (*p* < 0.001). The mean difference was 2.36, with greater variability among AS patients, likely reflecting disease heterogeneity (Table 2, Fig. 1).

**Table 2:** DXA T-score comparison between patients with AS and primary OP

Group	N	Mean (SD)	Median	Range	T-test (p)	Mann–Whitney U (p)	Mean Diff (95% CI)	Effect Size/Notes
AS	23	−2.24 (2.58)	−1.70	−9.8 to + 3.0	t(45) = 3.94 (p < 0.001)	U = 86.5 (p < 0.001)	2.36 (1.15–3.57)	Higher heterogeneity in BMD
Primary OP	24	−4.60 (1.36)	−4.55	−7.6 to −2.9				Significantly lower BMD

*AS* ankylosing spondylitis, *OP* osteoporosis. Data include mean (SD), median, range, independent samples *t*-test, Mann–Whitney *U* test, and mean difference with 95% confidence interval. *p* < 0.05 is considered statistically significant.

### Disease activity in AS

Among AS patients, the mean BASDAI score was 3.32 (SD = 1.39), with a median of 3.20, indicating moderate disease activity. BASDAI scores ranged from 0.5 to 6.5, with an interquartile range (IQR) of 2.30 (25th percentile) to 4.08 (75th percentile), reflecting that 50% of patients reported moderate symptoms within these values.

### Serum TRACP5b levels across groups

**Fig. 2:**
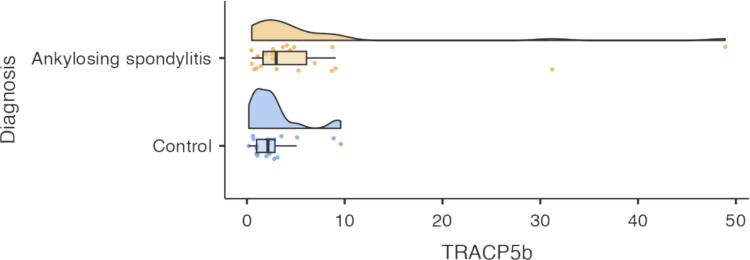
Violin plot showing TRACP5b levels in AS patients and healthy controls

Mean TRACP5b levels were higher in AS patients (mean 6.8 ± 11.1 ng/mL; range 0.5–48.9) compared to healthy controls (mean 2.7 ± 2.5 ng/mL; range 0.2–9.6), but this difference was not statistically significant (*p* = 0.111). The AS group also showed greater variability, potentially reflecting disease activity or treatment differences (Fig. 2).

### Graphical distribution

The violin plot illustrates a tighter distribution of TRACP5b values in controls, consistent with low bone resorption activity in healthy individuals. In contrast, AS patients showed a broader distribution with a rightward skew, indicating that while many patients had levels like controls, a subset exhibited markedly elevated values, suggesting increased bone turnover in some cases.

### Diagnostic performance

**Fig. 3:**
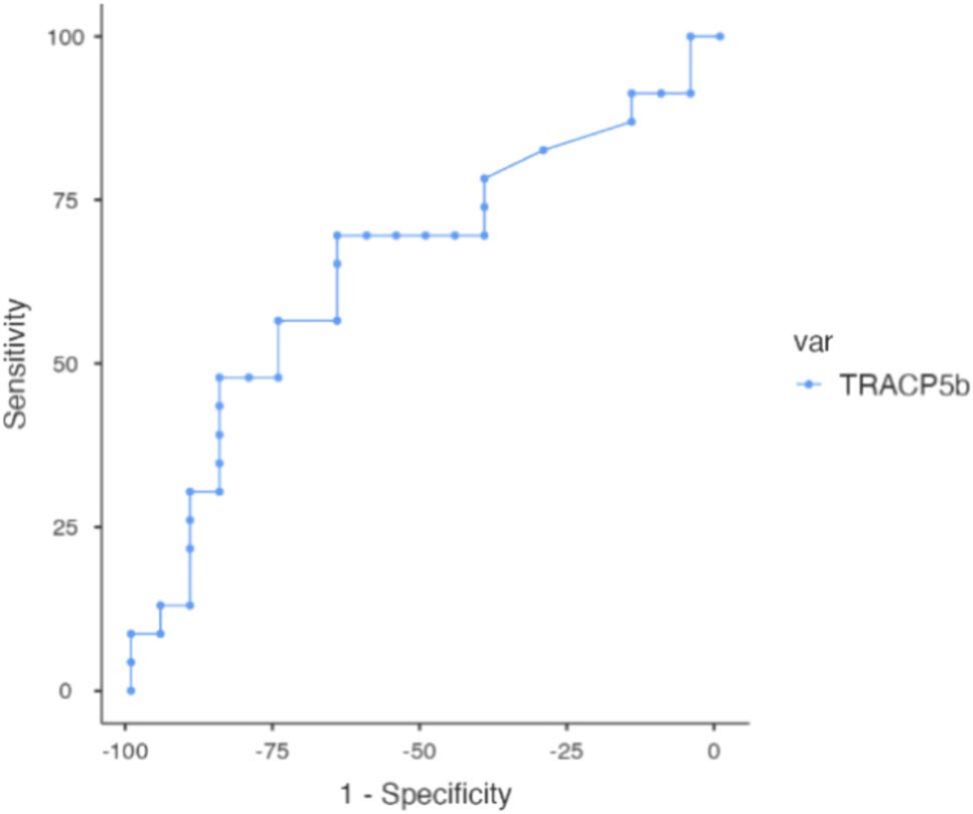
ROC curve showing the diagnostic performance of TRACP5b for detecting secondary OP in AS patients

ROC curve analysis demonstrated a moderate ability of TRACP5b to detect secondary OP in AS patients, with an area under the curve (AUC) of 0.653. A cut-off value of 2.6 ng/ml provided the best balance of sensitivity (69.57%) and specificity (65%). Othe tested thresholds (2.4–3.7 ng/ml) produced comparable diagnostic metrics (Fig. 3).

### Correlations of TRACP5b and DXA with clinical variables in AS and primary OP

In AS patients, TRACP5b levels correlated significantly with age (*r* = 0.534, *p* = 0.009) and BASDAI scores (*r* = 0.427, *p* = 0.042), but not with DXA T-scores, disease duration, or ESR. DXA T-scores correlated positively with disease duration only (*r* = 0.434, *p* = 0.039) (Table 3). In primary OP patients, no significant correlations were found between TRACP5b, DXA scores, or age; all associations were weak and non-significant; therefore, they are not shown in tabular form.

**Table 3:** Correlation of TRACP5b levels and DXA T-scores with age, disease duration, BASDAI and ESR in patients with AS

		TRACP5b	DXA
**Age**	Pearson's r	0.534	0.190
	Df	21	21
	*P* value	0.009**	0.384
	N		23
**Disease Duration**	Pearson's r	0.173	0.434
	Df	21	21
	*P* value	0.429	0.039*
	N	23	23
**BASDAI**	Pearson's r	0.427	0.191
	Df	21	21
	*P* value	0.042*	0.384
	N	23	23
**DXA scan**	Pearson's r	0.287	—
	Df	21	
	*P* value	0.184	
	N	23	
**ESR**	Pearson's r	0.077	−0.195
	Df	21	21
	*P* value	0.728	0.372
	N	23	23

*TRACP5b* Tartrate-Resistant Acid Phosphatase 5b, *DXA* Dual-energy X-ray Absorptiometry, *BASDI* Bath Ankylosing Spondylitis Disease Activity Index, *ESR* Erythrocyte Sedimentation Rate. Pearson’s correlation coefficient (*r*) and *p* values are shown. * *P* < 0.05, ** *P* < 0.01.

### Comparison across AS treatment regimens

No significant differences were observed across AS treatment groups (non-biologic therapy, anti-TNF, or IL-17A inhibitors) regarding age, gender, smoking status, disease duration, laboratory markers, BMD and BASDAI scores. The IL-17A group showed the lowest median DXA T-score (−2.1), TRACP5b level (2.2) and BASDAI score (2.3), compared to higher values in anti-TNF and non-biologic groups (TRACP5b: 3.5 and 3.7; BASDAI: 3.2 and 3.9, respectively), although these differences were statistically non-significant.

## Discussion

In this study, patients with primary OP were significantly older and predominantly females, aligning with well-established risk factors such as age, female sex, and reduced sex hormones [18]. Conversely, the AS group showed marked male predominance and a significant higher smoking prevalence, consistent with ankylosing spondylitis epidemiology where male sex and smoking are established risk factors linked to increased disease activity and structural progression [19–22]. The near absence of smokers in the OP group may reflect gender-based lifestyle differences or greater health awareness among older female patients, reinforcing smoking’s role as a modifiable risk factor in AS populations.

Disease duration was significantly longer in AS group compared to primary OP group. This likely reflects the chronic inflammatory nature of AS, which often begins early in life and progresses over decades, impacting bone health and contributing to disease-related OP [23, 24]. In contrast, primary OP is often diagnosed later in life and may remain undetected until the occurrence of a fracture, explaining the shorter reported duration. These findings validate the clinical distinction among the studied cohorts [4].

Although we found no significant difference in reported history of fragility fracture between patients with AS and those with primary OP, this should be interpreted with caution. Large studies confirm that AS patients have a higher risk of fractures, especially vertebral fractures, due to low BMD, chronic inflammation and structural spinal changes [25]. For example, a 2015 national study showed AS patients have a fivefold increased risk of spine fractures compared to general population [26]. Similarly, a 2023 cross-sectional study found that nearly half of AS patients had low BMD, and 16% had at least one vertebral fracture [27]. However, vertebral fractures in AS are often underdiagnosed because they may be asymptomatic or masked by chronic back pain or misdiagnosed as acute back pain from their usual inflammatory pain [28]. Likewise, in primary OP, many fragility fractures remain undetected as shown in a 2021 database study which found that only a minority of patients receive screening or treatment before or after a fracture [29]. Together, these findings suggest that our rates of reported fractures may reflect underdiagnosis rather than true absence of risk, highlighting the need for better screening and vertebral imaging in both groups.

In this study, primary OP patients had significantly lower DXA T-scores than AS patients, that is consistent with literature showing that syndesmophytes can mask true bone loss in AP DXA in AS. Klingberg et al. [24] and Fitzgerald et al. [30] demonstrated that lumbar AP DXA underestimates BMD in AS due to syndesmophytes and spinal hyperostosis, which can result in falsely normal or elevated BMD values despite underlying trabecular bone loss. These structural changes predominantly involve posterior spinal elements and vertebral margins, contributing to overestimation of lumbar spine BMD. In contrast, lateral and volumetric DXA which focus primarily on vertebral trabecular bone and exclude posterior elements and syndesmophytes, provide more accurate assessment of osteoporotic changes in AS.

In our study, we found that TRACP5b levels were higher in patients with AS compared to healthy controls, but this difference did not reach statistical significance. This pattern is consistent with recent studies that report mildly elevated TRACP5b levels in AS without consistently significant differences compared to controls. For example, a large 2024 cohort study similarly found no significant differences, highlighting this marker’s limited ability to distinguish AS from non-AS populations in cross-sectional analyses [7]. Several factors may explain our finding: substantial variability within the AS group including a few patients with unusually high TRACP5b values, likely widened the standard deviation and masked true differences, the modest sample size also limited the power to detect subtle effects, and the considerable overlap in lower-range TRACP5b values between groups further reduced its discriminative potential. While some of this variation may reflect random noise, it may also point to genuine biological heterogeneity within the AS population. This suggests TRACP5b could still have value for identifying subgroups with more active bone resorption. Larger studies with careful stratification by disease activity, treatment exposure, and inflammatory status are needed to clarify whether TRACP5b can meaningfully support bone health assessment in AS.

Our DXA findings along with TRACP5b levels, supports the previous studies indicating that chronic inflammation in AS contributes to systemic bone involvement and loss. Collectively, these results highlight inflammation-driven skeletal fragility in AS [31]. Taken together, our results suggest that increased osteoclast activity in AS does not always match BMD changes seen on DXA, and elevated TRACP5b may identify heightened osteoclast activity and inflammation-driven bone remodeling even when BMD changes are modest, reinforcing the importance of using both biochemical and imaging markers for more complete assessment.

Notably, in AS patients, TRACP5b showed significant positive correlations with age (*r* = 0.534, *p* = 0.009) and disease activity measured by BASDAI (*r* = 0.427, *p* = 0.042), but not with BMD, disease duration or ESR. Limited research has explored the relationship between TRACP5b and AS disease activity; some existing studies indicate that TRACP5b correlates with disease activity markers and structural damage in AS, although correlations with BASDI are often inconsistent or modest. For example, Toussirot et al. [14] reported strong correlations between TRACP5b and laboratory inflammatory markers, but not with BASDAI scores. Similarly, Çınar et al. [27] reported no significant correlation between disease activity score and BMD, reinforcing the complex relationship between disease activity and bone loss in AS.

The broad range of BASDAI scores in our patients reflects the varied disease burden and levels of control within the AS group, likely influenced by differences in disease duration, treatment and comorbidities. This variation may help explain why some correlations were only moderate.

In this study, we evaluated the diagnostic performance of TRACP5b for identifying secondary OP among patients with AS compared to healthy controls. The ROC analysis showed that TRACP5b has a moderate predictive value, with AUC of 0.653, indicating limited discriminative power as a stand-alone marker. The optimal cut-off value of 2.6 ng/ml balanced sensitivity and specificity reasonably well, suggesting it could serve as a practical threshold for early detection and assessment of OP risk in AS patients. These findings imply that while TRACP5b can contribute to decision-making, it should be interpreted alongside other clinical, biochemical, and imaging markers rather than used alone. This is consistent with previous reports that highlight TRACP5b as a strong bone resorption marker in specific metabolic contexts. For example, a broad retrospective study in a Chinese population reported that TRACP5b was positively correlated with OP risk in newly diagnosed type 2 DM patients after adjusting for age and BMI [32], and Gossiel et al. [33] who highlighted its utility for monitoring treatment responses in OP patients receiving anti-resorptive therapy. Together, these observations support the idea that diagnostic performance of TRACP5b may vary depending on the underlying cause of bone loss. In AS, given the moderate AUC in our analysis, future studies should further investigate its value in combination with BMD measurements and other markers to improve the diagnostic accuracy and guide management.

In our study, no significant differences were observed among AS treatment groups regarding age, gender, smoking status, disease duration, or laboratory markers. TRACP5b levels and BMD values also did not differ significantly between groups. Although the IL-17A inhibitor group exhibited lower median BASDAI scores, TRACP5b levels, and slightly reduced median DXA T-scores compared to other groups, these differences were not statistically significant, with wide interquartile ranges indicating substantial individual variability. These findings are consistent with a research in the TURKBIO cohort that revealed that AS patients exhibited similar disease activity levels with comparable biologic and non-biologic treatment approaches [34]. Also, Nechvátal et al. [35] findings were in line with ours, they revealed that no significant difference in BASDAI score was observed in both conventional and biological treated groups of patients. The lack of significant differences in BMD may reflect that short- to medium-term biologic therapy does not markedly influence bone turnover or density in AS, possibly due to chronic progression of bone loss. The numerically lower BASDAI score in the IL-17A inhibitor group suggests a trend toward improved disease activity control, but the statistical non-significance underscores the need for larger sample sizes or longitudinal studies to more thoroughly evaluate therapeutic effects.

This study has some limitations. Although the minimum sample size required by a priori power calculation for the primary outcome was achieved, the overall cohort remains relatively modest, which may have limited the detection of smaller effect sizes, the performance of smaller subgroup analysis, and the generalizability of the findings. Therefore, the absence of statistical significance in some comparisons may reflect limited precision rather than a true lack of biological relevance. The cross-sectional, single-center design precluded evaluation of longitudinal changes in TRACP5b levels and BMD over time and limited causal inference. Additionally, serum vitamin D levels, sun exposure and vitamin D supplementation were not systematically assessed, which may represent an unmeasured confounder influencing BMD interpretation. Furthermore, DXA measurements in AS patients may be affected by syndesmophytes, potentially underestimating actual bone loss. Larger, multicenter longitudinal studies are therefore needed to validate the utility of TRACP5b as a monitoring tool and to clarify its prognostic value in predicting fracture risk among AS patients.

Despite these limitations, our findings contribute to understanding the role of bone turnover markers in inflammatory OP and highlight the need for integrated approaches combining imaging and biochemical markers for comprehensive bone health assessment in AS.

## Conclusion

This study demonstrated that serum TRACP5b levels are elevated in AS patients compared to healthy controls, reflecting increased osteoclast activity associated with chronic inflammation. While TRACP5b showed moderate diagnostic value for secondary OP in AS, it did not prove its usefulness for primary OP, highlighting the different pathophysiological mechanisms between inflammatory and degenerative bone loss. The lack of strong correlation with BMD further supports the need to interpret TRACP5b alongside imaging findings for more accurate assessment of skeletal health. Although no significant differences were observed in TRACP5b levels across AS treatment regimens, our findings emphasize the potential role of combining biochemical markers with DXA to enhance early detection and monitoring of bone fragility in AS. Future large-scale, multicenter longitudinal studies are needed to validate these results and clarify the utility of TRACP5b as part of comprehensive OP management in patients with chronic inflammatory arthritis.

## Supplementary Information

Below is the link to the electronic supplementary material.

Supplementary file1 (PPTX 40 KB)

## Data Availability

The datasets generated and analyzed during the current study are available from the corresponding author on reasonable request.
