# Supplementary material for: Diagnostic utility of serum TRACP5b for secondary osteoporosis in ankylosing spondylitis: a comparative cross-sectional study with primary osteoporosis and healthy controls
Source: Clin Rheumatol. 2026 Feb 17;45(4):2277–85. doi: 10.1007/s10067-026-07966-7 (PMC12979343; doi:10.1007/s10067-026-07966-7)
Supplement: Supplementary file 1 — Supplementary file1 (PPTX 40 KB) [file 10067_2026_7966_MOESM1_ESM.pptx]

## Slide 1
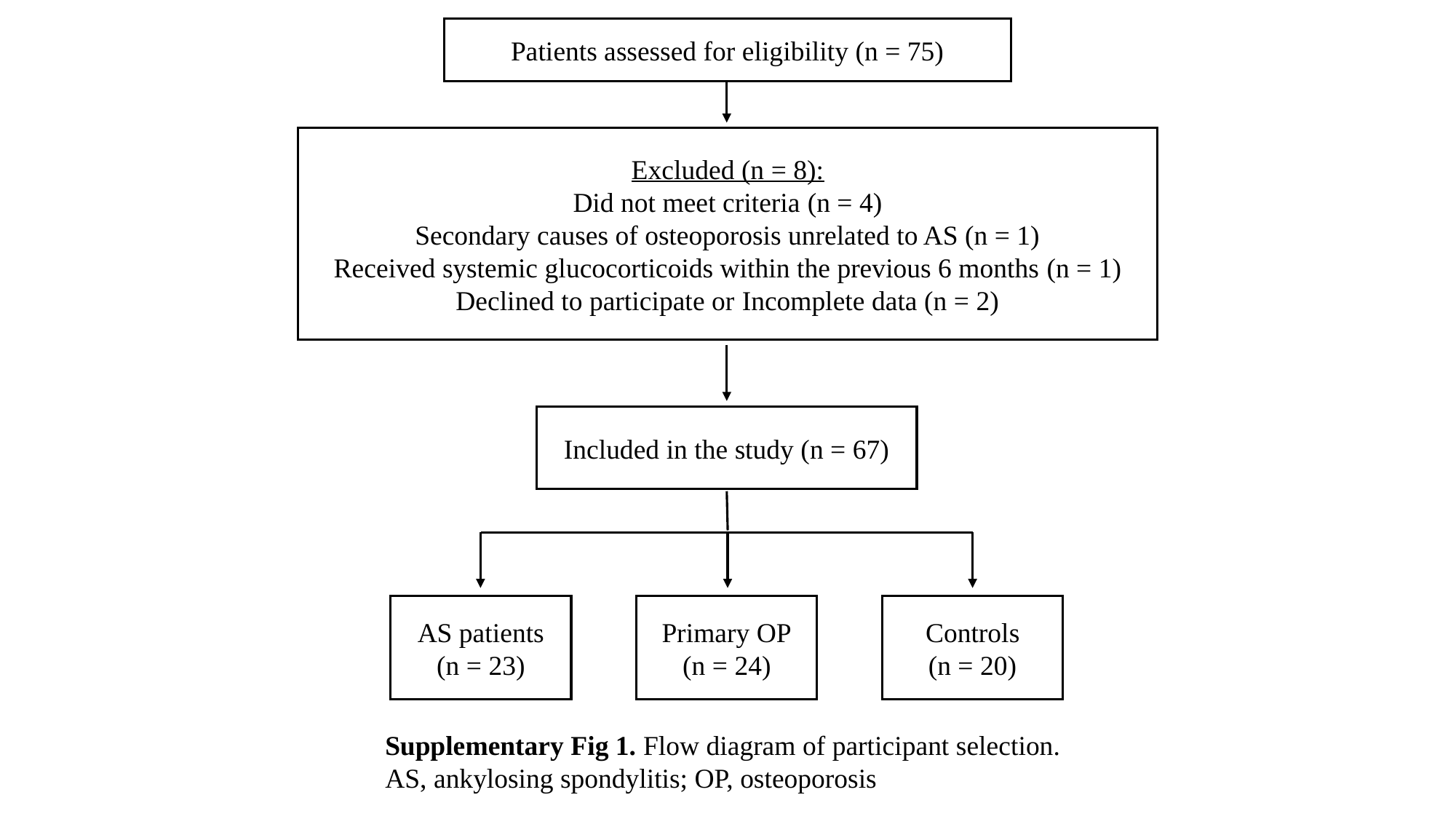

Patients assessed for eligibility (n = 75)
Excluded (n = 8):
Did not meet criteria (n = 4)
Secondary causes of osteoporosis unrelated to AS (n = 1)
Received systemic glucocorticoids within the previous 6 months (n = 1)
Declined to participate or Incomplete data (n = 2)
Included in the study (n = 67)
Primary OP
(n = 24)
Controls
(n = 20)
AS patients
(n = 23)
Supplementary Fig 1. Flow diagram of participant selection. AS, ankylosing spondylitis; OP, osteoporosis
